# Identification of Proteins with Potential Osteogenic Activity Present in the Water-Soluble Matrix Proteins from *Crassostrea gigas* Nacre Using a Proteomic Approach

**DOI:** 10.1100/2012/765909

**Published:** 2012-05-01

**Authors:** Daniel V. Oliveira, Tomé S. Silva, Odete D. Cordeiro, Sofia I. Cavaco, Dina C. Simes

**Affiliations:** Center of Marine Sciences, University of Algarve, Campus de Gambelas, 8005-139 Faro, Portugal

## Abstract

Nacre, when implanted *in vivo* in bones of dogs, sheep, mice, and humans, induces a biological response that includes integration and osteogenic activity on the host tissue that seems to be activated by a set of proteins present in the nacre water-soluble matrix (WSM). We describe here an experimental approach that can accurately identify the proteins present in the WSM of shell mollusk nacre. Four proteins (three gigasin-2 isoforms and a cystatin A2) were for the first time identified in WSM of *Crassostrea gigas* nacre using 2DE and LC-MS/MS for protein identification. These proteins are thought to be involved in bone remodeling processes and could be responsible for the biocompatibility shown between bone and nacre grafts. These results represent a contribution to the study of shell biomineralization process and opens new perspectives for the development of new nacre biomaterials for orthopedic applications.

## 1. Introduction

Bone is formed by mineral deposition (calcium phosphate in the form of hydroxyapatite) in an organic matrix, and materials composed of calcium phosphate are potential substitutes for bone grafts as they connect with the living tissue; however, these materials are very fragile. Some molluscs contain a material designated by nacre (mother-of-pearl), a natural biomaterial formed by a regular superimposition of polygonal aragonite calcium carbonate tablets 0.5 mm thick arranged in a brick-wall structure embedded in a thin organic cell-free matrix layer comprising less than 2% of total weight. Nacre is considered a natural ceramic composite with the same density of bone, and several *in vitro* and *in vivo* studies have shown that nacre has excellent biocompatibility and osteogenic properties suggesting its use as a bone substitute in orthopedic surgery [1–6].

 Nacre is usually composed by polygonal aragonite platelets, each one composed by CaCO_3_ crystals, with a thickness of 0,5 *μ*m and a width of 5–20 *μ*m, being organized in a columnar structure with a brick and mortar arrangement. The organic shell matrix constitutes between 0,1–5% (w/w) of nacre shell weight, is believed to be essential for control of shell biomineral formation, and contains several macromolecules including polysaccharides (mostly chitin), proteins (both water-insoluble framework proteins and water-soluble proteins), and glycoproteins that are present in both inter and intracrystalline locations within the nacre structure. This organic matrix secreted by the external mantle epithelium constitutes about 5% (by weight) of nacre and the remaining 95% correspond to the aragonite platelets [2, 3, 7–11]. Interestingly, there are reasons to believe that although nacre is functionally and structurally similar between different species of bivalves (for instance, they all contain chitin and display carbonic anhydrase activity), the specific protein composition is quite heterogeneous across species [12].

 The most recent studies using a proteomic approach for identification of the proteins that constitute the extracellular calcifying matrix of mollusks shells only confirm the following: although dozens of different proteins from nacreous tissues have already been identified, there is seldom any significant sequence overlap between the nacre proteomes of different species [12–16].

*In vivo* studies suggest the use of nacre as a biomaterial compatible in bone repairing, since fragmented nacre from *Pinctada maxima* grafted onto sheep femurs was shown to stimulate bone repairing [4]. It is known that nacre possesses valuable properties in terms of strength, durability, and biologic interaction with the host's bone. It has been demonstrated that osteointegration of *Pinctada maxima* nacre implants of long duration (9 months) occurs without the insertion of fibrous tissues [1]. *In vitro* studies with three mammalian cell types: fibroblasts, bone marrow stromal cells, and osteoblasts [5, 6, 17] indicated that the water-soluble extract of *Pinctada maxima *nacre matrix displays an osteoinductor effect, suggesting the existence of at least one agent within the WSM (water-soluble matrix) with the ability to stimulate bone formation. *In vitro* studies with preosteoblastic mice cells (MC3T3-E1) also confirmed that low molecular weight molecules present in the nacre WSM obtained from *Pinctada maxima *shell stimulate the differentiation of the preosteoblasts into osteoblasts, denoting an inductive effect in mineralization [17, 18].

WSM is constituted by the water-soluble molecules of the nacre organic matrix of *Crassostrea gigas* although its exact composition (such as the identification and biochemical characterization of the agent or agents responsible for the *in vitro* osteoinductive effect) has not yet been achieved. The main purpose of this work was the characterization of the proteins contained in this matrix, as the complete proteome of the *Crassostrea gigas* nacre WSM has not yet been characterized.

## 2. Materials and Methods

### 2.1. Extraction of the Water-Soluble Matrix (WSM)

The oysters (8-9 cm in length) were obtained from a local hatchery of the Ria Formosa (Faro). The shells were thoroughly clean from adhering soft tissues and washed with tap water. Nacre was scratched from the inner shell layer of *C. gigas* oysters and lyophilized. After lyophilisation, nacre was ground to powder (particle size 80–100 *μ*m), and 100 g of it was suspended in 200 mL of Milli-Q water for 20 h at 4°C with constant stirring (450 rpm, Stirrer ES (VELP, Scientifica)).

 The suspension was centrifuged at 4000 rpm for 20 minutes at 4°C, the supernatant filtered through Whatman filter paper grade 4 (20–25 *μ*m pore) and stored in 2 mL aliquots at −20°C. The filtered supernatant extract obtained constitutes the nacre Water-soluble matrix (WSM) that was further analyzed.

### 2.2. Quantification and Purification of the WSM Extracts

The total protein present in the WSM extract was quantified with Quick Start Bradford Protein Assay kit (Bio-Rad) [19]. After quantification, several samples (1,76 mL, 500 *μ*g total protein) were prepared and then lyophilized. To obtain samples with low conductivity and remove ionic contaminants from our samples, we used the ReadyPrep 2-D Cleanup Kit (Bio-Rad, ref. 1632130) in order to purify the protein samples for 2DE analysis. The precipitates were then solubilized in IPG rehydration buffer (7 M urea, 2 M thiourea, 2% (w/v) CHAPS, 50 mM dithiothreitol (DTT), and 1% (v/v) IPG PharmaLyte pH 3–10 ampholytes).

### 2.3. Two-Dimensional Electrophoresis (2DE)

3 samples with 400 *μ*g, 280 *μ*g, and 250 *μ*g of protein, respectively, were prepared (these samples were prepared because there is a tradeoff between the quantity of protein on the strip and the amount of spot smearing, which results in gels with little protein for the abundant proteins and gels with more protein for the less-abundant proteins), and the samples diluted to a final volume of 210 *μ*L with the ReadyPrep rehydration buffer composed of 8 M urea, 2% CHAPS, 50 mM DTT, 0,2% (w/v) Bio-Lyte 3/10 ampholytes, and a trace of bromophenol blue. The samples were added to IPG strips ReadyPrep pH 4–7, 11 cm (Bio-Rad) using passive rehydration loading, overnight at 20°C. Isoelectric focusing (IEF) of proteins was performed using a four-step protocol (Step  1: a gradient from 0 V to 250 V over the course of 3 hours, Step  2: a gradient from 250 V to 1000 V over the course of 1 h 30 m, Step  3: a gradient from 1000 V to 3500 V over the course of 5 hours, Step 4: a step and hold at constant 3500 V during 1 h 13 min) and a Ettan IPGphor Cell (Amersham) at a maximum current of 50 *μ*A/strip at 20°C. Focused strips were stored at −20°C until separation by second dimension.

 Before the second dimension separation, the proteins on each IPG strip were reduced for 30 minutes with 2 mL of an equilibration buffer (EBI) [6 M urea, 2% (w/v) SDS, 0,375M (pH 8,8) Tris-HCl, 20% (v/v) glycerol and 2% (w/v) DTT (Bio-Rad)], and 14,81 *μ*L of DTT solution (2,7 mg/*μ*L). After the reduction step, the EBI was removed and the proteins on each strip alkylated for 30 minutes with 2 mL of an equilibration buffer (EBII) [6 M urea, 2% (w/v) SDS, 0,375M (pH 8,8) Tris-HCl, 20% (v/v) glycerol (Bio- Rad)] with 2,8% (w/v) of iodoacetamide.

 After the alkylation step, each strip was quickly washed with MOPS running buffer pH 7,7 (50 mM MOPS, 50 mM Tris, 0,1% (w/v), and 1 mM EDTA) and loaded on 13,3 × 8,7 cm Criterion XT gels (Bio-Rad) with the wells previously filled with an agarose solution for 2DE, composed of 0,5% (w/v) low melting point agarose, 25 mM Tris, 192 mM glycine, 0,1% (w/v) SDS, and vestigial quantities of bromophenol blue (Bio-Rad). Additionally, 8 *μ*L of SeeBlue Pre-Stained Standard (Invitrogen) were added to the gel where the strip containing 250 *μ*g of protein was loaded. Electrophoresis was then performed at 200 V constant voltage in a Criterion Cell (Bio-Rad). The gels were then rinsed with water 3 times for 10 minutes to remove any remaining SDS. Gels were placed in a solution of 50% (v/v) methanol and 10% (v/v) acetic acid for 15 minutes and then rinsed with water for 15 minutes. Staining was performed overnight with colloidal Coomassie brilliant blue G-250 (EZBlue (Sigma)) and then washed with deionized water.

### 2.4. Identification of the WSM Proteins by Mass Spectrometry (LC-MS/MS)

Protein spots were excised and stored in Eppendorfs tubes with 20 *μ*L of Milli-Q water at −80°C. Eight of these spots were selected and analyzed by LC-MS/MS at the Aberdeen Proteomics facilities.

 The proteins in the protein spots were reduced and alkylated (with DTT and iodoacetamide, resp.). Each reduced and alkylated protein was then subjected to tryptic digestion (using an autolysis-resistant modified trypsin) and the resulting peptides were extracted with formic acid and acetonitrile. The peptides were then analyzed in an UltiMate 3000 LC System (Dionex) coupled to a HCTultra PTM Discovery System (spherical ion trap) with a low-flow nebulizer (Bruker Daltonics). The liquid chromatography separation was performed using a PepSwift monolithic PS-DVB capillary column (200 *μ*m i.d. × 5 cm; Dionex), 2,0 *μ*L/min flow rate, and a linear gradient of acetonitrile. Peptide peaks were detected and deconvoluted automatically using DataAnalysis software (Bruker Daltonics). At the end of the run, mass lists in the form of Mascot Generic Files (.mgf) were created automatically.

 The mass lists generated in the previous step were then used as an input to Peptide Fragment Fingerprinting (PFF) searches on the nonredundant database of NCBI (NCBInr) using the Matrix Science webserver (http://www.matrixscience.com/). The search was performed on the NCBInr database assuming the carbamidomethylation of cysteine residues, the formation of double or triple charged peptides, allowing up to 1 missed cleavages, oxidation of methionine residues, carboxylation of glutamate residues, a 1,5 Da peptide mass tolerance and a 0,5 Da MS/MS mass tolerance and the taxonomy as “other Metazoa”. Whenever no significant results were obtained with the NCBInr database, searches in the EST_others database were performed afterwards. Due to the fact that ESTs are seldom properly annotated, the resulting sequences were then compared to the NCBInr protein sequences (using BLASTP, http://blast.ncbi.nlm.nih.gov/) in order to find proteins with significantly similar sequences. Refinement of identifications towards particular isoforms was confirmed by performing multiple alignments using MCOFFEE (http://www.tcoffee.org/). Additionally, automated detection of conserved domains and motifs was performed using InterProScan (http://www.ebi.ac.uk/Tools/pfa/iprscan/) and ELM (http://elm.eu.org/) [20].

## 3. Results

**Figure 1:**
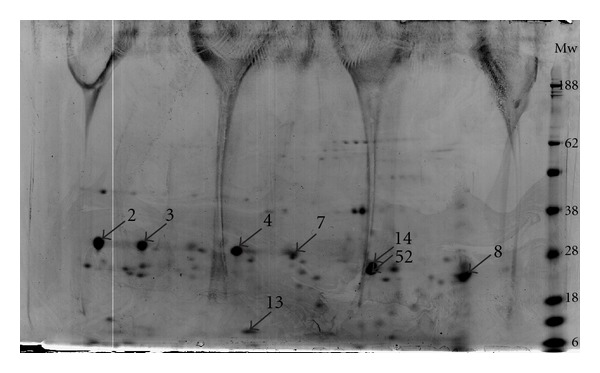
2DE gel (11 cm, pH 4–7, Bis-Tris 12%) showing all the protein spots detected in nacre WSM from *Crassostrea gigas*. Numbered spots represent proteins identified by mass spectrometry.

From the 3 electrophoresis gels performed, two were used to excise the spots for LC-MS/MS analysis and one containing the prestained molecular weight markers was used to estimate the molecular weight of the spots.  Figure 1 represents a 2D gel (pH 4–7 11 cm, Bis-tris 12%) of WSM *Crassostrea gigas* nacre where spots selected for mass spectrometry analysis are numbered. WSM 2, 3, 4, 7, 8, 13, 14, and 52 (Figure 1) represent the 2D protein spots selected for identification.

The WSM spots 2, 3, 4, 7, 8, 14, and 52 (molecular weight between 19 kDa and 38 kDa, Figure 1), shown to be well resolved in 2-DE gel, were also selected for sequence analysis because there were some reports that described a 19 kDa protein from oyster *Pinctada fucata*, denominated N19, having a role as negative regulator of pearl calcification [21]. Until now, N19 has only been identified in the water-insoluble matrix; however, it seemed relevant to see if any of these unidentified proteins with similar molecular weights could have some role in the biomineralisation processes.

 Recent works using two mineralogenic cell lines have shown that a protein-denominated p10 obtained from *Pinctada fucata* oyster, with a molecular weight around 10 kDa, could accelerate the nucleation of the calcium carbonate crystals, inducing the formation of aragonite, suggesting that this p10 protein could play an important role in nacre biomineralisation. The protein was also shown to induce osteoblast differentiation, since the results also showed increased ALP activity [22]. The conjugation of this data led us to further select the WSM 13 spot (Figure 1) for sequence by LC-MS/MS.

 After LC MS/MS analysis of the spots, the data obtained was processed with the MASCOT search engine as described in Materials and Methods section. Eight spots were identified with a high degree of certainty as homologous to certain EST sequences by peptide fragment fingerprinting (PFF). Identification was then performed by BLASTp alignment of obtained EST sequences (Table 1) against the NCBInr database, using the default settings (http://www.ncbi.nlm.nih.gov/blast/Blast.cgi). Significant matches (E-value < 0.05) were then confirmed by making sure identified peptides were present in the matched sequences, as well as by performing multiple alignments and domain/motif prediction analysis. In some cases, the same protein was identified in more than one gel spot of similar molecular mass (Figure 1, Table 1). These may represent a PTM such as phosphorylation or proteolysis.  Table 2 contains a summary of the proteins identified on the eight spots selected for analysis after the BLASTp alignment and complementary analyses.

## 4. Discussion

A total of four proteins present in the WSM nacre obtained from *Crassostrea gigas* were identified using a 2DE and LC-MS/MS approach, although one of them has already been identified in the insoluble fraction of *Crassostrea gigas* nacre matrix. After 2DE separation, selected spots (Figure 1) were identified by homology as three isoforms of Gigasin-2 (WSM 2 and WSM 3, WSM 4 and WSM 7, WSM 14 and 52) and cystatin A2 (WSM 8 and WSM 13). These proteins were found to be the most abundant in each of the excised spots, according to the respective emPAI (exponentially modified protein abundance index) values, which constitute a label-free estimate of the abundance of each protein within each spot [23].

One of the gigasin-2 isoform identified (corresponding to WSM 2 and WSM 3) has already been previously described by Marie et al. [24] as being present in the insoluble fraction of *C. gigas* nacre matrix, while the other two identified isoforms (corresponding to WSM4/7 and WSM 14/52) have not been previously identified. Gigasin-2 contains two conserved EGF domains, being somewhat homologous to Wnt inhibitory factor-1 (WIF-1) and tenascin C. Wnt inhibitor factor-1 is well described as an evolutionary conserved protein, member of the secretory Wnt modulators, which contains a single WIF domain that mediates Wnt direct binding, and five epidermal growth factor-like repeats [25].

Wnt proteins belong to a family of nineteen secreted glycoproteins, associated with the regulation of several development processes like embryogenesis, organogenesis, and oncogenesis [26–32]. The signalling of Wnt that results in tissue-specific activation of target gene transcription is initiated by the binding of Wnts ligands to Frizzled receptors and the coreceptor lipoprotein receptor-related protein 5/6 (LRP5/6). This metabolic pathway is also regulated by the secretion of antagonists that prevent ligand-receptor interaction, binding directly to the Wnt proteins or to the LRP5/6 component of the Wnt receptor complex [32]. These interactions activate multiple intracellular signalling cascades that include the canonical/*β*-catenin pathway which is the most studied Wnt signalling pathway. Wnts and its membrane receptor complex are known to be expressed in bone, and it is now widely established that this family of growth factors plays a central role in regulation of bone, bone remodeling, and bone regeneration [33–37].

Clinical results have identified in human, a link between bone mass and mutations in LRP-5 with loss of function causing osteoporosis pseudoglioma syndrome and gain of function leading to pathological thickening of bone [38, 39]. Genetic studies with knockout and transgenic mouse models for Wnt pathway components have also demonstrated that this signaling pathway modulates the most important osteoblast physiologic processes, including proliferation, differentiation, and bone matrix mineralization as well as inhibiting bone-resorbing osteoclast function (for reviews see [35, 37]).

WIF-1's role in Wnt signalling was first described in human retina with very well-conserved orthologous proteins also identified in *Xenopu*s and *Zebrafish *[40], and although only limited data are available on the role of the Wnt antagonist WIF-1 in osteoblasts, a recent study indicates that WIF-1 plays a role as a negative regulator of osteoblastic differentiation in mouse mesenchymal C3H10T1/2 cells *in vitro* [36].

Altogether the available information on canonical Wnt signaling as a bone formation regulator and the identification in nacre WSM (Figure 1) of proteins homologous to Wnt inhibitory factor-1 leads us to suggest that Wnt antagonists present in nacre may be related to the WSM nacre known osteogenic activity although further studies are necessary in order to understand the role of WIF-1 in bone development.

 Tenascins are a glycoprotein family associated with the organic extracellular matrix (ECM), that induce proliferation, differentiation and cellular migration [41–43]. Four members of this family (tenascins C, R, X and W) have been identified and characterized in vertebrates [44]. The basic structure of tenascins are composed by epidermal growth factor (EGF) repeats in the direction of the amino end, fibronectin type III domains and a globular fibrinogen domain in the carboxylic end. Differences in both the number and nature of EGF and fibronectin type III domains can be observed between different species [41, 42]. Tenascin C is a disulfide hexamer, with a cysteine-rich center, composed by subunits with a molecular mass of approximately of 200 kDa. These subunits can have different masses due to glycosylation [41]. In mammals, the subunits are constituted by 14.5 EGF repeats with 8 fibronectin type III domains present in all the tenascin C isoforms [45].

Tenascin C is abundant in ECM both during connective tissue and bone development [46]. It is also found in ECM during smooth muscle development and expressed in kidney cells [47]. Tenascin C favors osteoblast differentiation, increasing ALP activity, as well as *in vitro* synthesis of specific bone proteins [48]. It also promotes proliferation of fibroblasts [49], smooth muscle [50] and tumor cells [51]. The mechanism of action underlying Tenascin C effect on cells is still unknown and is currently being investigated. It was found that the EGF repetitions of this protein can bind the EGF receptors and activate them [52]. It has also been reported that Tenascin C is able to induce growth-stimulating mechanisms like the Wnt signaling pathway [53].

We can then assume that the identified Gigasin-2 proteins (which display homology to proteins such as WIF-1 and Tenascin C) might not only be one of the proteins responsible for the observed biocompatibility of nacre grafts in bone, but may also have a relevant role in shell mineralization of *Crassostrea gigas*.

The protein identification results obtained for spots WSM 8 and 13 were matched as having homology to Cystatin A2. Cystatins are a family of proteins well known as inhibitors of cysteine proteinases (for review see [54]), and this family is widely shown to be present in different organisms including animals and plants [55]. Besides this inhibitory common function, these proteins are organized in three different families with high structural diversity that range, in family 1 cystatins, from 11 kDa unglycosylated intracellular proteins lacking disulfide bonds, like cystatins A and B, through family 2 cystatin secreted proteins, presenting with slightly higher molecular masses (13-14 kDa), sometimes glycosylated and containing disulfide bonds, as, for example, cystatin C. A third family includes much higher molecular weight complex proteins comprising three family-2 cystatin domains, disulfide bonds, and carbohydrate groups. Nonetheless, all these cystatin superfamily members include a conserved motif, namely, a Gln-Xaa-Val-Xaa-Gly motif in the central region of the polypeptide chain (where Xaa is any amino acid) [56, 57] that our results confirmed to be present in WSM 8 and 13. Cystatins mainly interact with the cysteine proteases papain and the mammalian cathepsins B, H, K, L, and S and are also known to inactivate cysteine proteinases released by invading microorganisms and parasites [57–59]. The presently identified cystatin A2 isoform could preclude that cystatin B may also be present in nacre since these proteins are structurally very similar. The identification in oyster and Manila clam of ESTs with sequence similarity to cystatin B reinforces our hypothesis [60–62].

Cystatin A has been also suggested to have a defense function against exogenous pathogens [57–59]. On the other hand, cystatin B is broadly distributed on cells and tissues and is considered a general cytosolic inhibitor, protecting cells against proteolytic degradation by cathepsins. The presence of cystatin A2 in *C. gigas *nacre may have a protective role in the organism since cystatins A inhibit cysteine proteinases from invading microorganisms and parasites. Interestingly, the effect of cystatin C on calvarial bone formation was examined in *ex vivo* and *in vitro* culture mouse systems, and the results showed that this protein stimulates osteoblast differentiation and bone formation [63, 64]. Also cystatin B may help regulating bone resorption by blocking the activity of cathepsin K in osteoclasts [65].

Cysteine cathepsins can be found in all living cells and constitute a wide protease family for which a wide range inhibitors including cystatins have been published and used (for review see [66]). Cathepsin K is a well-described cysteine protease mainly involved in collagen type I degradation in bone and teeth and highly expressed in human osteoclasts [67, 68]. Cathepsin K knockout mice were shown to develop osteopetrosis caused by impaired osteoclastic bone resorption, and these results confirmed the role of these proteases in bone degradation processes. In fact, several studies report the effect of cathepsin K inhibitors on the reduction of biomarkers of bone resorption both *in vitro* and *in vivo* [69, 70]. More recently, ovariectomized cynomolgus monkey, a known postmenopausal osteoporosis model, was subject to a long-term treatment with a cathepsin K inhibitor and showed an unexpected stimulatory effect on periosteal bone formation [71].

The inhibition of serine and cysteine proteases, namely, K proteinase, by the WSM nacre fraction was previously observed by Bédouet et al. reinforcing our belief that cystatin A2 might be one of the proteins involved in bone resorption [72]. Recently, other protease inhibitors have been identified by Marie et al. on the nacre of various mollusks [12, 14, 15], and they propose that the role of these protease inhibitors could be the protection of the organic matrix against degradation by exopeptidases, but they can also suggest a role in the remodeling of the shell matrix. We believe that these proteases identified in other mollusks may also be present in the *C. gigas* nacre WSM extract, since we still have unidentified protein spots in our 2DE, with molecular weights similar to the previously described [14, 15]. In addition, spots WSM 8 and 13 show a different gel migrations behavior corresponding to different molecular weights, and this fact is consistent with the possibility that spot WSM 13 is a fragment of the identified spot WSM 8 protein, since its theoretical molecular weight obtained from the EST is identical to the result obtained from spot WSM 8. Although type1 cystatins have a molecular weight similar to spot WSM 13, we believe that, in this case, the identified cystatin has a molecular weight correspondent to 22 kDa. This observation is consistent with known literature since there are plant cystatins that have a similar molecular weight, raising the hypothesis that oyster cystatins might have evolved similarly (with the augmentation of the molecular weight explained by intragenic duplication effects) [55, 73, 74].

In conclusion, during this work, four proteins (three Gigasin-2 isoforms and one cystatin A2) were identified and proven to be present in WSM of the nacre of the oyster *Crassostrea gigas*. These are thought to be involved in bone remodeling processes, and some could have a role in the biocompatibility shown between bone and nacre grafts. In the future, these proteins and also the remaining proteins present in the WSM of nacre are expected to be identified and their osteogenic and osteoinductive properties tested in cell lines to verify if it is their combined effect that induces the bone remodeling or if they have an effect on their own. Unsurprisingly, only one of the four proteins identified in this work was previously identified, at the protein level, in the nacre of bivalves (specifically, in the insoluble fraction of *G. gigas* nacre matrix), which underlines the need for further studies elucidating the protein content of bivalve nacre matrix.

## Figures and Tables

**Table 1:** Significant results from the mass spectrometry (LC-MS/MS) through the analysis on the MASCOT web search engine.

Spot	EST accession number	Database	Taxonomy	Mw (KDa)/pI exp.	Mw (KDa)/pI theo.	Mowse score	Range (%)	High score peptide	Peptide *e*-value
WSM 2	gi|164584724	EST_others	*Crassostrea gigas*	30/4,2	24,195/6,45	388	44%	TTVVSTHNYNQFGPR	0.00024
	gi|164584223				23,488/5,24	301	26%	DSIMDVSCVHTNSFQGSTK	1.4*e* − 07
	gi|164576479				25,192/8,52	142	14%	SIYVGDSFLDENFDIK	2.3*e* − 06
WSM 3	gi|164584724	EST_others	*Crassostrea gigas*	29/4,6	24,195/6,45	359	44%	TTVVSTHNYNQFGPR	0.034
	gi|164584223				23,488/5,24	236	26%	DSIMDVSCVHTNSFQGSTK	0.0036
	gi|164576479				25,192/8,52	87	7%	SIYVGDSFLDENFDIK	0.0001
WSM 4	gi|164582888	EST_others	*Crassostrea gigas*	28/5,2	23,109/5,28	317	28%	GFFSMSDTVHSVNCVYEAR	7.9*e* − 06
	gi|168810136				17,317/4,89	112	13%	NNGLCDYFGR	0.26
	gi|115724273	NCBInr	*Strongylocentrotus purpuratus*		31,155/9,14	46	3%	IEVSDLDMGR	0.037
WSM 7	gi|164582888	EST_others	*Crassostrea gigas*	27/5,6	23,109/5,28	258	28%	LFELEIPHR	0.055
	gi|168810136	EST_others/NCBInr			17,317/4,89	189	28%	CPPGYQGYDCGLDSSTISSSADCR	0.00086
WSM 8	gi|164570244	EST_others	*Crassostrea gigas*	22/6,9	19,248/8,94	192	31%	LPADDAGTGIFEPISYK	0.017
	gi|14581004		*Crassostrea virginica*		21,663/9,29	80	6%	TQVVAGTNYFVK	0.00078
WSM 13	gi|164570244	EST_others	*Crassostrea gigas*	10/5,3	19,248/8,94	258	25%	LPADDAGTGIFEPISYK	1.8*e* − 06
	gi|14581004		*Crassostrea virginica*		21,663/9,29	84	6%	TQVVAGTNYFVK	0.00035
WSM 14	gi|164582378	EST_others	*Crassostrea gigas*	25/6,2	14,929/7,59	338	57%	GICFNGISCYCPEDYIGTK	2.6*e* − 06
	gi|152813529		*Crassostrea virginica*		10,815/5,76	71	22%	AYRPLNFNGEMFLK	3.2
WSM 52	gi|164582378	EST_others	*Crassostrea gigas*	25/6,2	14,929/7,59	257	56%	GICFNGISCYCPEDYIGTK	4.8e-05

**Table 2:** Putative identity of the proteins identified by mass spectrometry (LC-MS/MS). Hits with the lowest E-value are represented which are proteins with the biggest homology to the peptides obtained from LC-MS/MS. The emPAI (Exponentially Modified Protein Abundance Index) value offers approximate, label-free, relative quantitation of the proteins in a mixture based on protein coverage by the peptide matches in a database search result [23].

Spots [EST Accession Number]	Putative identity	emPAI score
WSM 2 [gi|164576479]	Peptidylprolyl isomerase B/cyclophilin B	0,28
WSM 2 [gi|164584223]	Gigasin-2	0,70
WSM 2 [gi|164584724]	Gigasin-2	1,47
WSM 3 [gi|164576479]	Peptidylprolyl isomerase B / cyclophilin B	0,13
WSM 3 [gi|164584223]	Gigasin-2	0,70
WSM 3 [gi|164584724]	Gigasin-2	1,47
WSM 4 [gi|115724273]	Putative serine/threonine-specific protein kinase	0,11
WSM 4 [gi|164582888]	Gigasin-2 like	1,25
WSM 4 [gi|168810136]	Gigasin-2 like	0,43
WSM 7 [gi|164582888]	Gigasin-2 like	0,97
WSM 7 [gi|168810136]	Gigasin-2 like	0,71
WSM 8 [gi|14581004]	PLCPI = cysteine proteinase inhibitor/cystatin A (stefin A)	0,16
WSM 8 [gi|164570244]	Cystatin A2	0,62
WSM 13 [gi|14581004]	PLCPI = cysteine proteinase inhibitor/cystatin A (stefin A)	0,16
WSM 13 [gi|164570244]	Cystatin A2	0,62
WSM 14 [gi|152813529]	Similar to Potassium channel subfamily K member 9	0,32
WSM 14 [gi|164582378]	Gigasin-2 like	4,19
WSM 52 [gi|164582378]	Gigasin-2 like	1,28
